# BioS2Net: Holistic Structural and Sequential Analysis of Biomolecules Using a Deep Neural Network

**DOI:** 10.3390/ijms23062966

**Published:** 2022-03-09

**Authors:** Albert Roethel, Piotr Biliński, Takao Ishikawa

**Affiliations:** 1 Department of Molecular Biology, Institute of Biochemistry, Faculty of Biology, University of Warsaw, 02-096 Warsaw, Poland; roethel.albert@gmail.com; 2 College of Inter-Faculty Individual Studies in Mathematics and Natural Sciences, University of Warsaw, 02-097 Warsaw, Poland; 3 Institute of Informatics, Faculty of Mathematics, Informatics and Mechanics, University of Warsaw, 02-097 Warsaw, Poland; pt.bilinski@uw.edu.pl

**Keywords:** deep neural network, feature vector, protein, protein fold classification

## Abstract

Background: For decades, the rate of solving new biomolecular structures has been exceeding that at which their manual classification and feature characterisation can be carried out efficiently. Therefore, a new comprehensive and holistic tool for their examination is needed. Methods: Here we propose the Biological Sequence and Structure Network (BioS2Net), which is a novel deep neural network architecture that extracts both sequential and structural information of biomolecules. Our architecture consists of four main parts: (i) a sequence convolutional extractor, (ii) a 3D structure extractor, (iii) a 3D structure-aware sequence temporal network, as well as (iv) a fusion and classification network. Results: We have evaluated our approach using two protein fold classification datasets. BioS2Net achieved a 95.4% mean class accuracy on the eDD dataset and a 76% mean class accuracy on the F184 dataset. The accuracy of BioS2Net obtained on the eDD dataset was comparable to results achieved by previously published methods, confirming that the algorithm described in this article is a top-class solution for protein fold recognition. Conclusions: BioS2Net is a novel tool for the holistic examination of biomolecules of known structure and sequence. It is a reliable tool for protein analysis and their unified representation as feature vectors.

## 1. Introduction

The era of structural biology began with the application of X-ray crystallography and nuclear magnetic resonance spectroscopy (NMR) to resolve the structure of biomolecules, such as nucleic acids and proteins. Recently, another powerful tool has been added to the arsenal of structural biologists, cryo-electron microscopy (cryo-EM), which has vastly accelerated the collection of three-dimensional structures of biomolecules and their complexes, achieving even atomic-level images of proteins at the resolution of 1.20–1.25 Å [1,2]. The largest repository of such data is the Protein Data Bank (PDB) [3], which contains proteins of both known and unknown function. With fast developing methods for resolving protein structures and rapid increase in the number of protein structures in the PDB, the need for automatic tools for structural analysis and annotation has also increased. Here, we present a novel deep learning-based bioinformatic tool called the Biological Sequence and Structure Network (BioS2Net). BioS2Net is a neural network designed for studying biomolecules of known structures represented as point clouds, where each point is represented by 3D spatial coordinates and can hold additional information, such as structural, physicochemical, and evolutional features.

BioS2Net is composed of four main parts: (i) a sequence convolutional extractor, (ii) a 3D structure extractor, (iii) a 3D structure-aware sequence temporal network, as well as (iv) a fusion and classification network. Our approach allows the extraction of both structural and sequential information embedded in protein point clouds and then merges extracted information together to deliver robust predictions.

**Figure 1:**
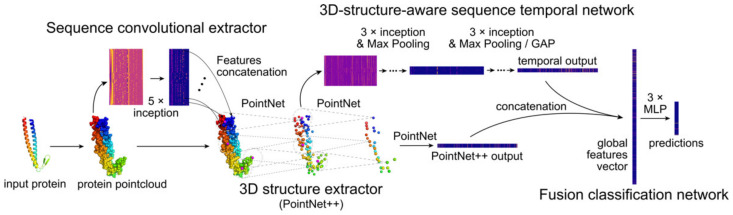
Summary of the BioS2Net architecture. Structural data obtained from a PDB-style file serve as a basis for generation of a protein point cloud. Sequential data is analysed by a sequence convolutional extractor with five inception modules. After features concatenation with a point cloud, merged data are interpreted by 3D structure extractor (PointNet++) in a hierarchical way by running a series of simplified PointNets. From the first set abstraction layer arises the 3D structure-aware sequence temporal network which takes as an input 512 points with raw features as well as those learnt by both extractors. It consists of six inception modules, followed by Max Pooling or Global Average Pooling. From both PointNet++ and the temporal network feature, vectors are obtained. Finally, a global feature vector is extracted and MLP is used to perform the classification. For readability, two classification heads, which introduce auxiliary losses, are omitted. Feature concatenation, which takes place while creating an input to the temporal network, is also not shown.

Overall workflow of the BioS2Net is as follows. At first, we run the temporal convolutional neural network to extract contextual features for each point of a cloud. Then, we extract 3D structure information from the input point cloud augmented with features extracted in the first step. Then, we apply our 3D structure-aware sequence temporal convolutional network. We fuse information together to produce a global feature vector, which is finally used for protein fold classification. It is worth emphasising that each point in the input of the 3D structure-aware sequence temporal network is aware of its structural and sequential surroundings, thus significantly leveraging the learning capabilities of BioS2Net (Figure 1).

Recently, a point cloud has drawn a lot of attention as an object representation method for deep learning [4]. There are many applications that involve neural networks learning from point clouds, such as (i) 3D shape classification, (ii) 3D object detection, and (iii) 3D point cloud segmentation, which includes semantic segmentation, instance segmentation, and part segmentation. To address those problems, several approaches have arisen. Guo et al. [4] have distinguished four major types of point cloud-centred neural network architectures: (i) pointwise multilayer perceptron (MLP) networks, (ii) convolutional-based networks, (iii) graph-based networks, and (iv) data indexing-based networks.

One of the first point cloud and deep learning-based architectures is PointNet [5]. It takes a set of points from ℝ^n^ space as input and applies a neural network for 3D classification and segmentation. This approach is invariant to the order of points, as well as to linear transformations, such as rotations and translations.

PointNet learns features by applying MLP to each point. Then, the approach performs the transformation using the symmetric max pooling function to aggregate information from each point and obtain a global feature vector. This allows the network to become invariant to the order of points. Finally, in case of classification problem, the global feature vector is mapped into output prediction using ordinary MLP layers.

There is a very limited number of papers which take advantage of representing molecules, such as proteins, as point clouds in the context of neural networks, without employing points voxelization. DeFever et al. [6] implemented PointNet in the modelling of molecular structures, although they focused rather on identifying local environments in molecular simulations. In the same work, the assessment of protein surface hydrophobicity was carried out by predicting whether a water molecule was localized in a hydrophobic or hydrophilic environment and projecting these predictions onto the protein surface.

Another approach based on PointNet was shown by Benhabiles et al. [7], which focuses on addressing the protein shape indexing problem. The authors used points representing a protein surface as inputs to the network and applied PointNet solely as a feature vector extractor. Weights in their PointNet were taken from the model pre-trained on generic, manmade objects, which share very limited similarity to the structure of protein surfaces. Additionally, unlike our solution, voxelization on point clouds was applied.

Recently, Toomer [8] applied the deep graph convolutional neural network (GCNN) to predict protein functional sites, claiming competitive results as compared to other approaches. However, he used several physicochemical features only to include more information about the atoms, thus not using the full potential of such an architecture. A similar solution was implemented by Nguyen et al. [9], where GCNN was used to model chemical bonds between atoms within chemical compounds and drug candidates, while the protein was represented as a sequence of amino acids without any additional information.

To summarise our approach in the context of the above-mentioned works, here we propose a novel deep neural network architecture that extracts both the sequential and structural information of biomolecules, and we use PointNet++ [10], the successor of PointNet, as the data representation model for structural analysis.

## 2. Results

### 2.1. Classification

The learning capabilities of this network have been verified by predicting protein folds, based on structural, physicochemical, and evolution-based information. It should be stressed here that it is not simply yet another approach to solve the protein fold recognition (PFR) problem [11,12,13,14,15,16]. The architecture presented in this work is rather a flexible tool for squeezing sequence- and structure-based data of biological structures into a feature vector that represents a protein in a reliable way. This can be further used for tasks such as PFR, as presented in this paper, but BioS2Net might be applied to solve other problems involving structural and sequential features of biomolecules.

**Figure 2:**
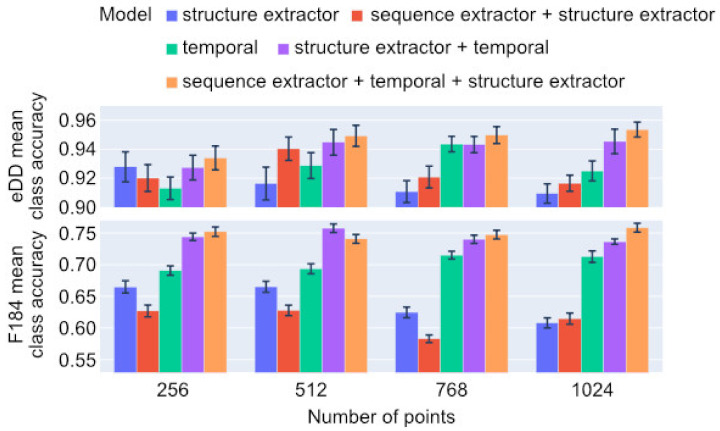
Ablation study on eDD and F184 datasets: mean class accuracy with standard deviation with regard to the number of sampled points using 20 algorithm executions.

We performed a classification on two widely used datasets: the eDD, consisting of 3397 proteins and 27 folds, and the F184, consisting of 6451 proteins and 184 folds. Our network achieves 95.4% mean class accuracy for the former and 76% for the latter with a full model trained on 1024 input points (Figure 2). Moreover, our network is able to properly classify protein folds even with a very limited number of atoms. Our full model with 256 input points achieves only a 2% accuracy drop on the eDD dataset, while the number of atoms was reduced four times. This property is highly essential from the perspective of involving our network in other applications due to the fact that biological structural data significantly vary in size and quality, so tools for studying them should be invariant to data incompleteness. Further analysis of the accuracy of our approach with respect to the number of input atoms is presented in Supplementary Figure S1.

Our full approach, including sequence, structural, as well as temporal networks, achieves 75.9% on the F184 and 95.4% on the eDD datasets using 1024 points and outperforms the remaining models on both datasets. To understand the impact of various elements on the accuracy of our approach, we performed the ablation study and analysed the mean class accuracy over models with various numbers of sampled points (Table 1). The most significant part of BioS2Net is the 3D structure-aware sequence temporal network, followed by the structural extractor, and finally the sequence extractor. When each is removed from the full model, the accuracy decreases by 7.94%, 3.29%, and 0.58%, respectively (Table 1). This confirms our assumption that sequential analysis of proteins is very important but misses crucial 3D structure information. Thus, the combination of both significantly improves recognition capabilities.

The temporal network performs better than PointNet++, which suggests that the sequential data might be more important than 3D protein structure in protein fold classification and/or that PointNet++ is suboptimal for the structural analysis of biomolecules. However, it is highly important to emphasise that PointNet++ is able to manage unordered sets of points in contrast to temporal networks. Nonetheless, the full approach combining the above-mentioned approaches outperforms the mean class accuracy, which confirms the effectiveness of BioS2Net (for more details, see Supplementary Tables S3 and S4).

**Figure 3:**
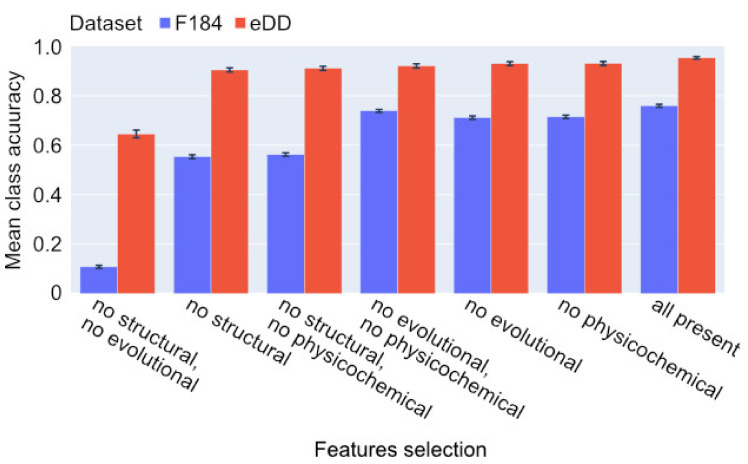
Impact of selected features on test accuracy. The number of points in the input are the same as those from the best full model from the particular dataset (1024 points for each). Mean taken from the last 20 epochs after the model reached a plateau. Whiskers represent standard deviations of test accuracy measures.

We then studied the impact of selected features on the model’s accuracy. Our experimental results confirm that the combination of both structure- and evolution-based features is crucial to obtain high classification accuracy (Figure 3). Mean top three class accuracy is provided in Supplementary Figure S2.

### 2.2. Embedding Protein Structures into ℝ^2^

**Figure 4:**
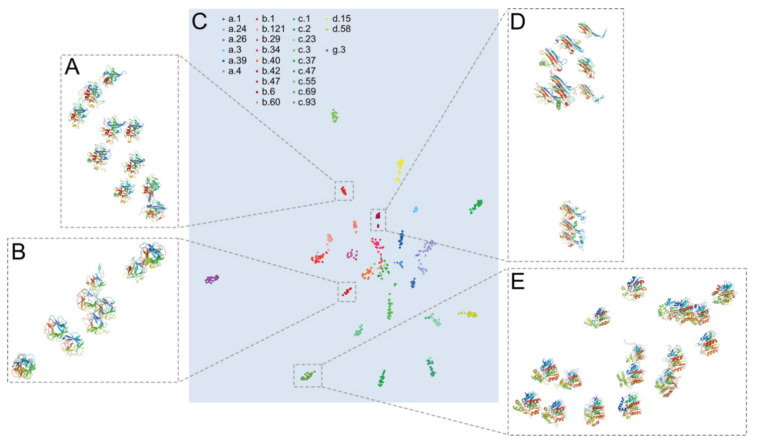
t-SNE embedding of global feature vectors retrieved from test proteins from the eDD dataset onto an ℝ^2^ plane. Proteins from the same structural class (such as all α proteins) are shown with similar colours (panel (**C**)). Side panels (**A**,**B**,**D**,**E**) show zoomed proteins from b.47, b.42, b.6, and c.69 folds, respectively, which are set within dashed rectangles on panel (**C**). All proteins within each group were structurally superimposed over one central protein of that group using PyMOL software [18], which was also used for rendering protein images.

In order to better understand what was learnt by our model, we extracted global feature vectors for proteins from the eDD test set. Then, we applied a T-distributed stochastic neighbor embedding (t-SNE) approach [17], which is a machine learning algorithm for dimension reduction and visualization, to present proteins on an ℝ^2^ plane (Figure 4).

We noticed that after t-SNE proteins with similar structures (belonging to the same fold) were placed next to each other, while proteins of different structures (belonging to different folds) were placed far away. We argue that BioS2Net is capable of mapping input point clouds to feature vectors, which seems to be in accordance with the structural division of proteins provided by SCOP. Despite the fact that learning was performed to obtain a fold classification, BioS2Net was able to represent proteins in such a way that feature vectors from proteins of the same structural class were located near each other. This also suggests that distinguishing structural classes in SCOP [19] is well-motivated. t-SNE embedding of the F184 dataset is shown in Supplementary Figure S3.

One can think of this embedding as an alternative or extension of PDB-Explorer [20], but instead of mapping proteins to shape space, we mapped them to sequence and structure space. Additionally, in contrast to PDB-Explorer, our approach allows for acquiring more clustered groups of proteins, which might be more desirable in some cases, such as PFR.

## 3. Discussion

In this study, we present a novel, extendable and flexible tool for studying biomolecules with known and well-defined sequences and structures. The idea of representing biological molecules as points clouds has not been widely exploited until now. There are only a few references to this approach in the literature in the context of neural networks [8,21] despite its having enormous potential, especially in cases of extensive and dynamic progress in point cloud learning [4]. To demonstrate the capabilities of our BioS2Net architecture, we trained it to recognise protein folds based on point clouds with rich biological information. Our experiments confirm the discriminative power of our representation and show that it can represent biomolecules in a reliable manner.

We argue that our approach could be useful for supporting the manual as well as automatic classification of newly solved protein structures in the SCOPe database. However, in this case, one can face the problem of classification to ca. 1200 of folds and ca. 2000 superfamilies. This may be solved by training hierarchical classifiers to properly predict folds and subsequently superfamilies. We believe that SCOPe may substantially benefit from our solution since its automatic annotation is mainly based on a simple BLAST algorithm [22] that is run against the SEQRES-based sequences from SCOP and SCOPe databases [19]. This approach does not involve any structural information. Additionally, to maintain a satisfactory level of error rate, rigorous conditions are set (further consideration is needed only for BLAST alignment with an E value less than 10^−4^). Moreover, BLAST is ineffective for domains with low sequence similarity to other proteins in SCOPe but which have high structural-level similarity. Such an approach causes a huge decrease in the number of domains being further analysed. In the face of the constantly increasing number of entries being deposited in PDB and limited human resources, a new approach is strongly advisable. BioS2Net’s accuracy obtained on the eDD dataset compared to results achieved by previously published methods indicates that the algorithm described in this report provides a top-class solution for protein fold recognition (Table 2).

Besides the methods listed in Table 2, the structure alignment methods represented by DALI and its stand-alone version, DaliLite, enables protein classification based on the structural similarities between the query structure and the one matched to it from the database [29]. The performance of the latest DaliLite v.5 is the best among competitors for protein classification at the fold- and superfamily-level [30]. The protein fold recognition benchmark has not been performed on DaliLite with the datasets used by us; thus, a direct comparison of accuracies would be inappropriate. However, together with the development of the ultra-fast computational method for protein structure search [31], structure alignment-based methods in the protein fold classification problem will become more and more accurate and efficient.

**Figure 5:**
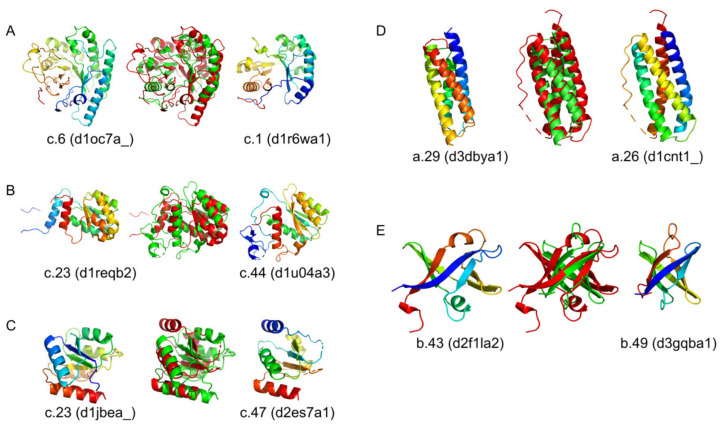
Examples of proteins from two distinct folds sharing a similar overall structure. Five different examples are shown in panels (**A**–**E**). In the middle of every panel, there is a structural superimposition of two proteins that are displayed on the left and right sides. The fold of every protein, as well as its stable domain identifier, are provided underneath the rendered images. For clarity, superimposed proteins are coloured with green and red. All pairs were detected by analysing t-SNE embedding, as presented in Figure 4.

Interestingly, after training our model, we recognised pairs of folds classified in SCOPe as distinct which, according to our output data, could be reclassified as belonging to a common metagroup (Figure 5). All examples represent overall structural similarities within the SCOPe class, which is not surprising, because folds categorised in the same class share secondary structures. The pairs of structures shown in Figure 5 differ in terms of the folding of backbones, although the overall 3D structures are similar enough to be identified by BioS2Net as belonging to the same group. Such proteins may fulfil a common function, for example, providing an interaction surface for other molecules, including proteins. An example of such structural convergence of the protein surface landscapes has been delivered by the comparison of small β-barrel SH3 and OB superfolds [32,33]. These structures are topologically distinct, yet overall 3D structures are close enough to play similar biological roles in nucleic acid metabolism [34].

Sadowski and Taylor reported that some members of distinct SCOP folds share identical topological descriptions [35]. In this analysis, topologies of protein folds were first represented as TOPS cartoons [36] and then transformed into topology strings which were finally clustered. Within clustered domains, there were c.2 and c.23 folds, among others. Strikingly, our classification made by BioS2Net located some members of c.2 and c.23 folds very close to each other on the t-SNE embedding. Taking these findings into account, we assume that additional fine-tuning of the annotation process of newly characterised protein structures (for instance, adding another meta-classification group) might be beneficial for the scientific community.

On the other hand, it should be noted that proteins with structurally distinct folds may be involved in the same biological activity [37]. Thus, although the current protein fold classification, such as SCOPe, is based on structural features, alternative approaches should also be considered.

The origin of such approaches may be found in the ideas behind Enzyme Commission numbers for the classification of enzymes or Gene Ontology. These classifications are made according to the function of biomolecules regardless of their 3D structures. Although Petrey and Honig have even suggested the avoidance of protein classification due to the multifunctionality and structural diversity of proteins [38], a recent report by Fontove and Del Rio clearly showed that the development of machine learning techniques enables the integration of structural and functional classifications of proteins [39].

BioS2Net can obviously be generalised and applied to other structured biological molecules, such as RNAs, peptides, or small chemicals, e.g., potential drugs. In general, our approach and its components are applicable wherever there is a well-defined sequence and/or structure. We hypothesise that, using confocal microscopy, one can visualize fluorescently labelled chromosome territories within cell nuclei [40]. Such stacked images can be represented as a point cloud and be fed into a PointNet-like network. As chromosome territories tend to be quite stable as well as cell- and tissue-specific [41], it might be useful to detect specified abnormalities (such as chromosome aberrations) or help to classify cells as normal or cancer-like [42].

Using BioS2Net, one can map complex, uneven, and comprehensive protein data onto small, fixed, and concise feature vectors. This information can subsequently be used for protein indexing, as shown by Benhabiles et al. [7], or similarity determination, i.e., by hierarchical clustering.

## 4. Materials and Methods

### 4.1. Architecture

Our approach consists of four main elements:

1. Sequence convolutional extractor;
2. 3D structure extractor;
3. 3D structure-aware sequence temporal network;
4. Fusion and classification network.

The first and third elements are temporal networks and they depend on the order of input points, whereas the second element extracts 3D structural patterns and is invariant with respect to the order of points. The last element combines the above-mentioned networks and makes the final prediction.

#### 4.1.1. Sequence Convolutional Extractor

The first part of our architecture is a convolutional extractor, which is designed to detect and extract sequence-based patterns and assign to each atom its sequential context. It takes raw point cloud with provided features as input. It is made up of five simplified 1D inception modules [43] with kernel sizes of 1, 3, 5, and 7 to detect patterns of varying length. The result is then concatenated with the input and together they are used as an input to the following modules (structure extractor and 3D structure-aware sequence temporal network).

#### 4.1.2. 3D Structure Extractor

The structural network is based on PointNet++. It is responsible for extracting structural patterns from the input and for their further mapping to a feature vector by abstracting selected local regions by their centroids. In our approach, we use three set abstraction layers, which map input point clouds to a new set of points with fewer elements but each of higher dimensionality so that each point becomes more general and reflects the wider structural context.

Each set abstraction layer consists of three sublayers: sampling layer, grouping layer, and PointNet layer. Having an input point cloud with N points and D associated features (including coordinates in metric space ℝ^3^), the sampling layer chooses a subset *S* out of *N* points as centroids. Then, for each centroid, the grouping layer selects K neighbouring points, resulting in S local regions. The output of this layer is of size *S* × *K* × *D*. Finally, we use PointNet, which applies MLP to each point in local regions and expands its feature vector to size *F*. For each local region, *K* × 1 max pooling is performed and a tensor *S* × *F* is obtained. This, in turn, results in representing each local region by its centroid. The last set abstraction layer has only one centroid, thus the output of this layer is a single 1024-dimensional feature vector. For more information, see Supplementary Table S1.

#### 4.1.3. 3D structure-Aware Sequence Temporal Network

The main goal of this component is to process a set of centroids that have merged both sequential and structural information and map them onto the feature vector. The temporal network starts from the output of the first set abstraction layer. It takes *M* centroids (*M* = 512 in our experiments) as an input, where each centroid has E associated features: from raw input and those learnt by sequence and 3D structure extractors. Then, it applies a series of 1D inception convolutions with a mix of filters of sizes 1, 3, 5, and 7, followed by 2 × 1 max pooling. The dilation rate is set to 3 to increase the receptive field. After the last convolutional layer, global average pooling is applied to obtain global feature vector representation. For more information, see Supplementary Table S2.

The main motivation for using this network is to combine the power of inception modules and the embedding of rich structural information learnt by PointNet++. Each input point of the temporal network contains information about its structural neighbourhood, as well as information from the atoms, which can be far away in the amino acid sequence. Therefore, the 3D structure-aware sequence temporal network is an important element of our approach.

#### 4.1.4. Fusion and Classification Network

At the final stage, the feature vector from the 3D structure extractor and the 3D structure-aware sequence temporal network are concatenated together as the global feature vector, which is then used as an input to our dense classification neural network. We use an auxiliary loss function for the 3D structure extractor and another auxiliary loss function for the temporal network to stabilize the training procedure. To properly manage updates of weights during the backpropagation, we use weighted auxiliary losses as follows: 0.5 for the prediction from the global feature vector, 0.35 for the prediction from the feature vector learnt by the 3D structure extractor, and 0.15 for the prediction from the feature vector learnt by the temporal network. See Supplementary Data, Section S1 for more details.

#### 4.1.5. Information Flow

The 3D structure extractor captures the 3D structural patterns of proteins but is invariant with respect to the order of points. Thus, we use the sequence convolution extractor to provide information about sequence-based patterns to the 3D structure extractor, which then provides information about 3D structural patterns to the 3D structureaware sequence temporal network. Finally, we fuse information from the 3D structure extractor and 3D structure-aware sequence temporal network, which provide complementary information to each other, to further improve the accuracy and allow network components to focus on different functions (Figure 1). Note that each architecture element has its advantages, but also limitations: sequence-based elements focus on sequence-based patterns and ignore structural information, whereas the structural component focuses on 3D structural patterns and ignores sequence-based information. In the proposed method, we combine the above-mentioned elements and their advantages to mutually strengthen them and create the superior classification method.

### 4.2. Datasetes

We performed protein fold classification based on structural, physicochemical, as well as evolutionary features. We evaluated our approach on two commonly used protein fold recognition (PFR) datasets: extended DD (eDD) [44] and F184 [14]. The former comprises 3397 domains from the SCOP 1.75 [19]. Proteins are classified into 27 folds, and each pair of proteins has less than 40% sequence identity. The latter is derived from SCOPe 2.06 [19]. The dataset contains 6451 domains from 184 folds with less than 25% pairwise sequence identity. In each group of folds, there are at least 10 proteins.

To test the accuracy of our neural network, we randomly divided each dataset into non-overlapping train and test sets. The number of proteins in the test set was 20 for groups with more than 100 proteins, and at least 30% of all proteins in the group have fewer than 30 observations. Linear and continuous interpolation was applied for the remaining groups.

There are six main classes of proteins distinguished in the SCOPe database: (a) all α proteins, (b) all β proteins, (c) α and β proteins a/b, (d) α and β proteins a + b, (e) multi-domains proteins, and (f) membrane and cell surface proteins and peptides. The eDD dataset contains proteins from the first four classes, whereas the F184 contains proteins from all of the above classes. In both datasets, two randomly selected amino acid sequences have small pairwise sequence identity, which makes the classification problem difficult.

### 4.3. Features

To provide comprehensive yet possibly minimal information about protein structure, physicochemical properties, and evolution, we represent each point, corresponding to a single atom of carbon, nitrogen, oxygen, and/or sulphur of a given protein, by 53 selected features. These features represent global (atomic-level) and local (amino acid-level) properties of proteins; see Table 3.

The features are as follows: three coordinates (x, y, z) in the Euclidean space. The fourth feature is the B factor, also called the temperature factor, which describes the displacement of the atomic position from an average position. For protein structures obtained by NMR or cryo-EM, there is no B factor annotation. Thus, for such structures, we used 0 as a B factor value to omit this component of the input data. Alternatively, algorithms for B factor prediction may be used [45,46]. The fifth parameter is the occupancy, which indicates whether there is an alternative conformation of a given atom. The fourth and fifth parameters derive from the PDB file and indirectly reflect local flexibility, which can be conservative within members of similar folds, especially if they are in the hydrophobic protein core [47].

The sixth attribute reflects the amino acid residue number in the primary structure of a protein in which the specific atom is located. It is calculated by dividing the atom number by the total length of a given protein sequence, and its value is in the range [0, 1]. The distance from the protein’s N-terminus defined in this way might seem to be redundant. However, small and local subsets of atoms are being selected during the training. Those atoms can capture structures or motifs which are comprised of amino acids from different fragments of the polypeptide chain. Thus, information about physical contact of amino acids or atoms from two distant parts of proteins might be crucial to recognise specific motifs and structures necessary for classification. Without this feature, our network would see only a bunch of atoms, which could be anywhere in the structure.

Moreover, we used two indicators describing whether an amino acid belongs to the α-helix of a β-sheet. We used the DSSP [48] to assess the secondary structure, and to limit the memory requirements, we divided structural classes from the DSSP into two groups: (i) 3_10_-helixes, α-helixes, and π-helixes, and (ii) β-sheets and β bridges. We assume that these two are the most important features after coordinates because they hold almost all the information about the secondary structure of proteins and are crucial to determine specific protein folds. The ninth property is the accessible surface area of amino acids calculated using the DSSP. Twenty consecutive logical features determine which amino acid each atom belongs to. This input information about amino acid sequence is highly useful for predicting folds and recognising motifs.

We also provided both physicochemical and evolution-based features proposed for protein fold recognition (PFR) by Dehzangi et al. [49]. We use the following four physicochemical features: charge (denoted as isoelectric point), polarity [50], polarizability [51], and hydrophobicity [52]. We also use twenty properties holding evolution-based information about the domain being learnt. We represent them by means of a position-specific scoring matrix (PSSM), which is of size *L* × 20, where *L* is the length of a given amino acid sequence. Each *ij* position of the PSSM specifies how probable is the substitution of the *i*-th amino acid in the sequence by the *j*-th amino acid from the set of 20 basic amino acids. The PSSM matrices are calculated by the PSI-BLAST algorithm [53] and were taken from Xia et al. [14] (http://yanglab.nankai.edu.cn/TA-fold/benchmark; accessed on 5 July 2021).

To normalise our data, we centred all proteins from both datasets and measured the distance of each atom from the origin. Next, we scaled the coordinates of all atoms, so that 95% of them were within a unit sphere. This was due to the fact that some of the proteins are non-globular and are thus treated as outliers in the dataset. This operation preserved the scale between any two distinct proteins within the dataset. Additionally, each of the non-coordinate features listed in Table 3 was normalised to be in the range [0, 1], with the exception of charge, which was normalised to be in the range [−1, 1].

We performed point cloud augmentation, which comprises random rotation in three axes by a random angle, random scaling, random translation, and slight point jittering. No point shuffling in any sequence of points was performed to preserve the order of atoms from PDB files.

### 4.4. Calculation of Accuracy

Simple accuracy was calculated as the number of correct classifications divided by all classifications. Mean class accuracy was an average of simple accuracies for each class. The latter was used to exclude the impact of unbalanced classes on model accuracy. All calculations were made only on the test set that was not exposed to the network during its training at any time. A test accuracy calculation was performed on the last 20 epochs when the model reached a plateau. This approach enables capture of the variability of the prediction accuracy on the stable model.

### 4.5. Computational Resources

Model parameters consumed up to 1 GB depending on the number of included components. The training was performed on the Entropy computation cluster (Faculty of Mathematics, Informatics and Mechanics, University of Warsaw, Warsaw, Poland) with the following GPU hardware: RTX 2080 Ti (MSI, Taipei, Taiwan), TITAN V, and TITAN X (NVIDIA, Santa Clara, CA, USA). The performance of these GPUs was enough to run the model.

The categorical cross-entropy cost function was minimised by Adam optimiser [54].

## 5. Conclusions

Here, we described BioS2Net, a novel tool for a holistic examination of biomolecules of known structure and sequence. We represent biomolecules as point clouds and embed sequential and structural contexts into each point. The possibility of adding to each point any feature on any structural level (such as atomic, amino acid, or even whole protein) makes our network highly extendable. Almost every component of BioS2Net can be run on its own and achieve sensible results. Moreover, this architecture is invariant with respect to the incompleteness of input data as well as to their random rotation and translation, as it learns hierarchically on local regions within the point cloud.

Results obtained in this work show that BioS2Net is a reliable tool for protein analysis and their unified representation as feature vectors. By comparing them, we also found potential structural convergence between several pairs of proteins with folds classified to distinct classes (Figure 5). Our solution might be used for global detection of such cases.

Feature vectors are useful in facing several bioinformatic problems. One of the most promising applications of BioS2Net is proper and reliable indexing of biomolecules for their comparison and analysis. For example, it will be possible to search a protein database with a query protein to find its equivalents that have similar features, such as presence of an intrinsically disordered region or a specific structural motif. Proteins are characterised not only in terms of 3D structure but also by physicochemical and evolutionary features. Additionally, in many cases, they significantly differ in size. For this reason, the above-mentioned database search would likely be a computationally intensive task. The application of BioS2Net for mapping proteins to fixed-size vectors for their further comparison seems to be an excellent alternative.

Note that this architecture can be easily extended to dense per-point regression and classification tasks, which could be useful, e.g., for the analysis of binding and interaction sites. Our approach is an alternative method to existing techniques, which are often based on atom voxelization, U-Net architecture, and 3D convolutions [21,55]. Notably, it may be further improved by employing one of the more sophisticated point cloud architectures reported by Guo et al. [4] or by including more discriminant features indicated in a variety of PFR-oriented approaches.

## Figures and Tables

**Table 1:** Ablation study: mean class accuracy decreases over various models.

		Dataset	
BioS2Net without:	eDD	F184	Both
Sequence convolutional extractor	−0.65%	−0.52%	−0.58%
3D structure extractor	−1.91%	−4.68%	−3.29%
3D structure-aware sequence temporal network	−2.22%	−13.66%	−7.94%

**Table 2:** Comparison with the different strategies for solving the protein fold recognition problem on the eDD dataset.

Method	Accuracy	References
PFPA (2015)	92.6%	[23]
ProFold (2016)	93.2%	[24]
PHMM-DP (2016)	92.9%	[25]
Xia et al. (2017)	94.5%	[14]
MV-fold (2019)	94.8%	[26]
MT-fold (2019)	97.1%	[26]
Refahi et al. (2020)	91.2%	[27]
Qin et al. (2021)	93.5%	[28]
BioS2Net	95.4%	This work

**Table 3:** Features assigned to each point in the point cloud.

Index	Feature	Value	Feature Type	Feature Level
1	x	ℝ	Coordinate	Atomic
2	y	ℝ		
3	z	ℝ		
4	B factor	[0, 1]	Structural	
5	Occupancy	[0, 1]		
6	Distance from N-terminus	[0, 1]		
7	Is ⍺-helix	Boolean		Amino acids
8	Is β-sheet	Boolean		
9	Accessible area	[0, 1]		
10–29	Amino acid	Boolean		
30	Charge	[−1, 1]	Physicochemical	
31	Polarity	[0, 1]		
32	Polarizability	[0, 1]		
33	Hydrophobicity	[0, 1]		
34–53	PSSM	[0, 1]	Evolutionary	

## Data Availability

The source code of BioS2Net is available at https://github.com/tryptofanik/bios2net (accessed on 7 March 2022).

## Supplementary Materials

The following supporting information can be downloaded at: https://www.mdpi.com/article/10.3390/ijms23062966/s1.
