# Supplementary material for: BioS2Net: Holistic Structural and Sequential Analysis of Biomolecules Using a Deep Neural Network
Source: Int J Mol Sci. 2022 Mar 9;23(6):2966. doi: 10.3390/ijms23062966 (PMC8954277; doi:10.3390/ijms23062966)
Supplement: Supplementary file 1 [file ijms-23-02966-s001.zip › ijms-1578267-Supplementary materials.pdf]

BioS2Net: Holistic structural and sequentialanalysis of biomolecules using deep neural network Supplementary data

Albert Roethel, Piotr Biliński and Takao Ishikawa

S1. Detailed architecture description

Our architecture comprises of four components, which are:

1. sequence convolutional extractor,
2. 3D structure extractor,
3. 3D-structure-aware sequence temporal network,
4. fusion classification network.

S1.1. Sequence convolutional extractor

The sequence convolutional extractor is based on simplified inception module with 4 paths of operations (for filters of size 1, 3, 5 and 7). Each path consists of 8following convolutions with the same filter size. The outputs are concatenated(by channels) and give a tensor of size 1024 32, in which each point has information about its sequential surrounding. Then, the tensor is concatenated with the raw input. Thus, we obtain a tensor of size 1024 85, which is an input to the 3D structure extractor and 3D-structure-aware temporal network.

S1.2. 3D structure extractor

The main component of the 3D structure extractor is the PointNet++ with 3set abstraction layers. Each of them consists of sampling layer, grouping layer,and PointNet layer. Every set abstraction layer takes as an input N pointswith D features and produces a tensor with fewer $N' < N$ points, but having more $D' > D$ features. In more detail, the sampling layer chooses N' centroids from N points. The grouping layer chooses K neighbours for each of centroids using a sphere of radius R, resulting in N' local regions, each being $K \times D$ tensor. Then, each local region is directed to PointNet layer which applies MLP expanding feature vector from D to D'. Finally, K-MaxPooling is performed, resulting in abstracting each centroid by its surrounding

Supplementary Table S1: Specific summary of set abstraction layers in Point-Net++

Layer	*N*-number of input points	N′–number of centoids	K - number of neighbours	D` -final feature number of a single point	R - search radius	Output tensor sizes
First	1024	512	64	128	0.2	512 × 128
Second	512	128	128	256	0.4	128 × 256
Third	128	1	128	1024	-	1 × 1024

Last set abstraction layer chooses only one centroid. Its output is a single 1024-dimensional feature vector, which represents the entire protein (Supplementary Table S1).

S1.3. 3D-structure-aware sequence temporal network

Outputs of the first set abstraction layer (512 × 128) of 3D structure extractor are concatenated with respective features from raw input (512 × 53) and with those returned from the sequence convolutional extractor (512 × 32), resulting in 512 × 213 tensor. It is then sent to the 3D-structure-aware sequence temporalnetwork, which comprises of 6 inception modules each followed by Max Pooling layer with exception to the last layer which is followed by Global Average Pooling(Supplementary Table S2). All inception modules have filters of sizes 1, 3, 5, 7 to capture patterns of varying length. Their proportion is 2 : 3 : 2 : 1. The output of this part is a feature vector of size 1 × 1024

We emphasise that the idea standing behind this part of the architectureis that the input to the temporal network is a sequence of atoms which are aware of their sequential and structural surroundings. Thus, it provides rich and comprehensive information to the powerful temporal-based network.

S1.4. Fusion classification network

The final element of the architecture is the concatenation of features vectors from 3D structure extractor and temporal feature vectors to one vector, which is followed by classification layer. Output scores are calculated by applying three MLP layers of sizes 512, 256, and *C*, where *C* is the number of classification groups. After each fully connected layer, there is a dropout with drop rate equalto 0.6.

Supplementary Table S2: Specific summary of temporal network.

layer	output shape	kernel shape	filters fraction	dilation rate
inception 1	512 × 64	[1, 3, 5, 7]	[2, 3, 2, 1]	3
MaxPool	256 × 64	2	-	-
inception 2	256 × 128	[1, 3, 5, 7]	[2, 3, 2, 1]	3
MaxPool	128 × 128	2	-	-
inception 3	128 × 128	[1, 3, 5, 7]	[2, 3, 2, 1]	3
MaxPool	64 × 128	2	-	-
inception 4	64 × 256	[1, 3, 5, 7]	[2, 3, 2, 1]	3
MaxPool	32 × 256	2	-	-
inception 5	32 × 512	[1, 3, 5, 7]	[2, 3, 2, 1]	3
MaxPool	16 × 512	2	-	-
inception 6	16 × 1024	[1, 3, 5, 7]	[2, 3, 2, 1]	3
GlobalAveragePool	1 × 1024	-	-	-

Moreover, we use 2 auxiliary losses to stabilize training: one arises from the

PointNet++ and the other from the temporal feature vector. They both follow the same classification layer and the same loss function, which is the cross-entropy

S1.5. Further implementation and training details

We train our approach until reaching plateau (120 epochs without improve- ment), or 600 epochs. We use batch normalization after each convolutional layer. We also use weighted loss function to compensate under- and over- represented classes due to significant class imbalance problem. Learning rate decreased exponentially throughout training from 0.001 to 0.00001 in staircase mode. The categorical cross-entropy cost function was minimised by Adam op-timiser (Kingma and Ba, 2014). In all experiments, the batch size was equal to 32. The training was performed on RTX 2080 Ti, TITAN V, and TITAN X GPUs (NVIDIA). Model parameters consume up to 1 GB depending on the number of included components.

S2. Supplementary results

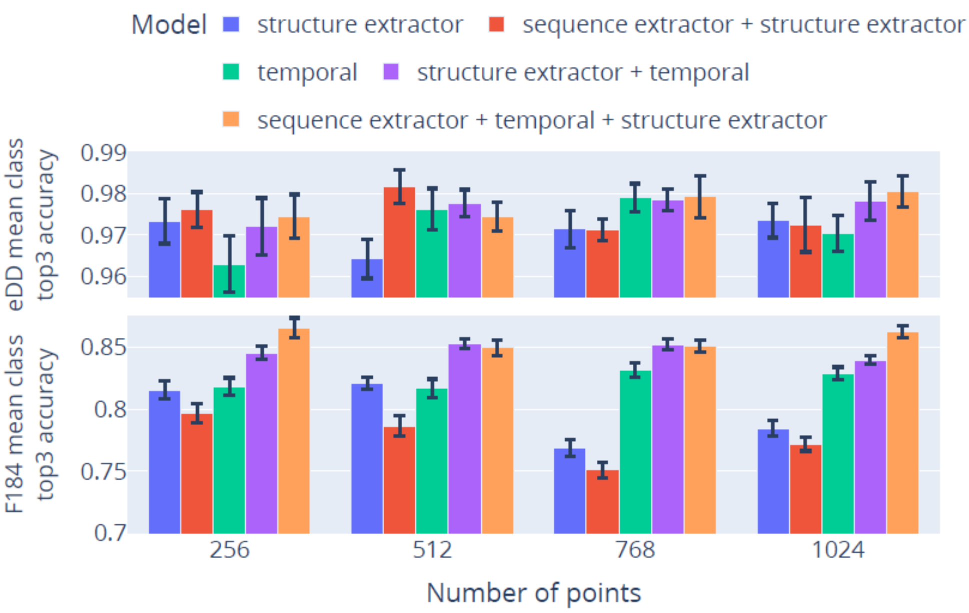

Supplementary Figure S1: Mean top 3 class accuracy for eDD and F184 datasetswith respect to the number of points in the input data and model used. Whiskersrepresent the standard deviation of mean top 3 class accuracy from last 20 epochs

Supplementary Table S3: Ablation study: influence of model components andnumber of points on accuracy.

dataset	model components			number of points			
	sequence extractor	structure extractor	temporal	256	512	768	1024
eDD		✓		92.79%	91.63%	91.07%	90.94%
	✓	✓		92.01%	94.03%	92.08%	91.65%
			✓	91.3%	92.87%	94.36%	92.5%
		✓	✓	92.73%	94.48%	94.32%	94.54%
	✓	✓	✓	93.39%	94.92%	94.98%	95.36%
F184		✓		66.5%	66.52%	62.47%	60.8%
	✓	✓		62.7%	62.78%	58.3%	61.49%
			✓	69.08%	69.35%	71.5%	71.28%
		✓	✓	74.41%	75.77%	74.01%	73.63%
	✓	✓	✓	75.23%	74.08%	74.74%	75.85%

Supplementary Table S4: Ablation study: min, max, and mean class accuracy decrease over various models.

model	F184			eDD			both		
	min	max	avg	min	max	avg	min	max	avg
full without temporal	-16.44%	-11.3%	-13.66%	-3.71%	-0.89%	-2.22%	-16.44%	-0.89%	-7.94%
full without structure extractor	-6.15%	-3.24%	-4.68%	-2.86%	-0.62%	-1.91%	-6.15%	-0.62%	-3.29%
full without sequence extractor	-2.22%	1.69%	-0.52%	-0.82%	-0.44%	-0.65%	-2.22%	1.69%	-0.58%
full without temporal									

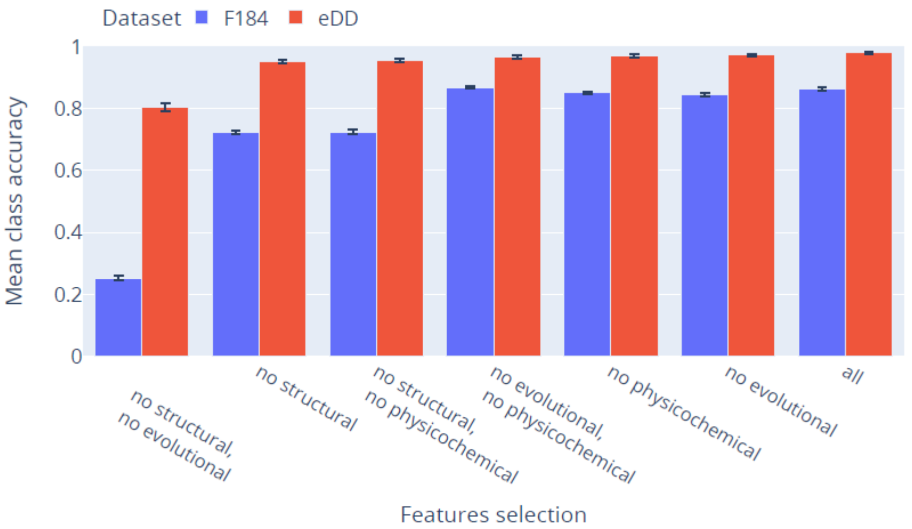

Supplementary Figure S2: Impact on mean top 3 class accuracy of selected fea-tures. The number of points in the input is the same as those from the best fullmodel from a particular dataset. Whiskers represent the standard deviation of mean top 3 class accuracy from last 20 epochs.

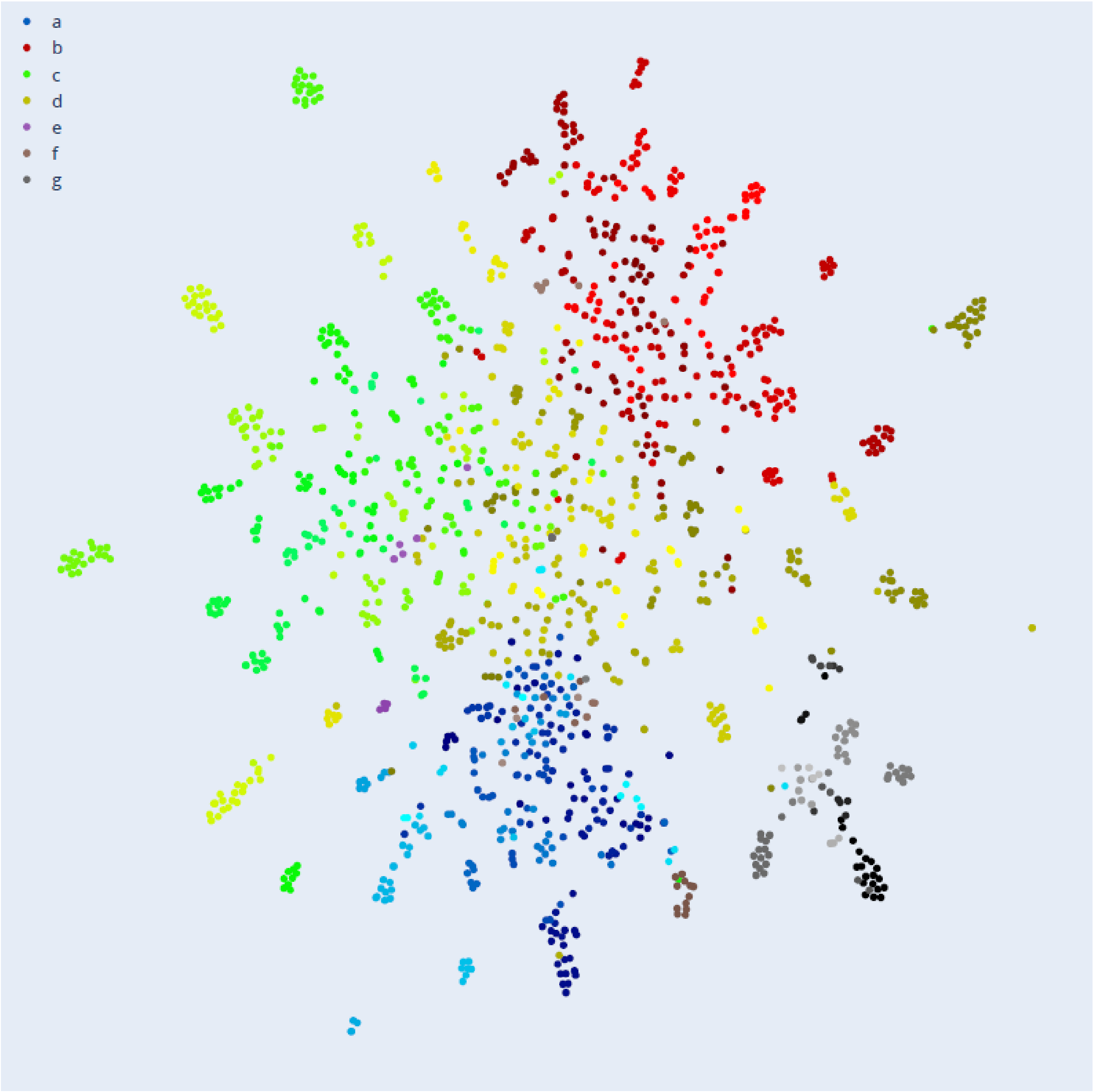

Supplementary Figure S3: t-SNE embedding of global feature vectors from F184dataset. Proteins from the same structural class (like all α proteins) are shown with a similar colour.

References

Kingma, D.P.; Ba, J. Adam: A Method for Stochastic Optimization. 2019. Available online: https://arxiv.org/abs/1412.6980 (accessed on 14 January 2022).
